# Adjusted Tumor Enhancement on Dual-Phase Cone-Beam CT: Predictor of Response and Overall Survival in Patients with Liver Malignancies Treated with Hepatic Artery Embolization

**DOI:** 10.3390/curroncol31060231

**Published:** 2024-05-29

**Authors:** Hooman Yarmohammadi, Fourat Ridouani, Ken Zhao, Vlasios S. Sotirchos, Sam Y. Son, Ruben Geevarghese, Brett Marinelli, Mario Ghosn, Joseph P. Erinjeri, Franz E. Boas, Stephen B. Solomon

**Affiliations:** Department of Radiology, Division of Interventional Radiology, Memorial Sloan Kettering Cancer Center, 1275 York Ave, New York, NY 10065, USA; ridouanf@mskcc.org (F.R.); zhaok@mskcc.org (K.Z.); sotirchv@mskcc.org (V.S.S.); sons1@mskcc.org (S.Y.S.); geevarr1@mskcc.org (R.G.); marinelb@mskcc.org (B.M.); mario.ghosn@aphp.fr (M.G.); erinjerj@mskcc.org (J.P.E.); ed.boas@gmail.com (F.E.B.); solomons@mskcc.org (S.B.S.)

**Keywords:** hepatic artery embolization, liver tumors, tumor enhancement

## Abstract

The aim of this study was to examine the value of tumor enhancement parameters on dual-phase cone-beam CT (CBCT) in predicting initial response, local progression-free survival (L-PFS) and overall survival (OS) following hepatic artery embolization (HAE). Between Feb 2016 and Feb 2023, 13 patients with 29 hepatic tumors treated with HAE were analyzed. Pre- and post-embolization, subtracted CBCTs were performed, and tumor enhancement parameters were measured, resulting in three parameters: pre-embolization Adjusted Tumor Enhancement (pre-ATE), post-embolization ATE and the difference between pre- and post-ATE (∆ATE). Treatment response was evaluated using the mRECIST criteria at 1 month. Tumors were grouped into complete response (CR) and non-complete response (non-CR) groups. To account for the effect of multiple lesions per patient, a cluster data analytic method was employed. The Kaplan–Meier method was utilized for survival analysis using the lesion with the lowest ∆ATE value in each patient. Seventeen (59%) tumors showed CR and twelve (41%) showed non-CR. Pre-ATE was 38.5 ± 10.6% in the CR group and 30.4 ± 11.0% in the non-CR group (*p* = 0.023). ∆ATE in the CR group was 39 ± 12 percentage points following embolization, compared with 29 ± 11 in the non-CR group (*p* = 0.009). Patients with ∆ATE > 33 had a median L-PFS of 13.1 months compared to 5.7 in patients with ∆ATE ≤ 33 (95% CI = 0.038–0.21) (HR, 95% CI = 0.45, 0.20–0.9, *p = 0.04*). Patients with ∆ATE ≤ 33 had a median OS of 19.7 months (95% CI = 3.77–19.8), while in the ∆ATE > 33 group, median OS was not reached (95% CI = 20.3-NA) (HR, 95% CI = 0.15, 0.018–1.38, *p = 0.04*). CBCT-derived ATE parameters can predict treatment response, L-PFS and OS following HAE.

## 1. Introduction

Hepatic artery embolization (HAE) is a well-established treatment for both primary and secondary hepatic malignancies [1,2]. Different imaging modalities, including immediate post-procedure CT scan and cone-beam CT (CBCT), have been used to document completion of embolization [3]. In addition to documenting completion of treatment, the pattern of retained contrast on immediate post-procedure CT after HAE has been used to predict treatment response in these patients [3]. In patients treated with conventional TACE (cTACE) or those treated with drug-eluting bead TACE (DEB-TACE), subjective or objective estimation of the degree and pattern of Lipiodol retention within the tumor immediately following treatment have been used to predict response [4,5]. Therefore, in HAE, cTACE and DEB-TACE, visual or subjective evaluation of the post-imaging scans, either on CT or CBCT, is the most popular method of evaluating completion of embolization and predicting overall response. These methods are non-automated and are solely dependent on the operator’s/reviewer’s assessment. Additionally, these methods are not precise when evaluating tumors that demonstrate inhomogeneous uptake. Therefore, there is a need to explore other objective imaging markers. The aim of this study was to examine the value of automated tumor enhancement parameters on CBCT obtained before and immediately after HAE in predicting treatment response, local progression free survival (L-PFS) and overall survival (OS).

## 2. Materials and Methods

### 2.1. Study Design

The Institutional Review Board approved this retrospective data analysis. Data of 13 patients with primary or liver-dominate metastatic disease treated between February 2016 and February 2023 were reviewed. Inclusion criteria included the following: 1. patients with primary or metastatic liver cancer that were treated with HAE; 2. pre- and post-treatment CBCT was available; 3. tumor enhancement parameters were measurable on the dual-phase CBCT. Patients treated with radio-embolization were excluded. Additionally, patients with poor-quality or incomplete dual-phase CBCT precluding accurate measurement of tumor enhancement parameters were also excluded. Patient records and labs were reviewed for age, gender, presence of cirrhosis and its etiology, type of liver malignancy, presence and type of prior treatments, baseline Model for End-Stage Liver Disease score, Child–Pugh score and Eastern Cooperative Oncology Group performance status. All the retrieved data were compiled into a Health Insurance Portability and Accountability Act-compliant database.

### 2.2. HAE Procedure

The HAE technique has been previously described [6]. Pre-embolization DSA and subtracted dual-phase CBCT were acquired with the catheter placed in the proper hepatic, right or left hepatic artery. Treatment was performed with either Embosphere (Merit Medical, South Jordan, UT, USA), Embozene (CeloNova BioSciences, Inc., San Antonio, TX, USA) or Poly-Vinyl Alcohol (PVA) (Cook Medical Inc., Bloomindale, IN, USA) particles until complete arterial stasis was achieved [7]. The definition of complete stasis was no forward flow in the treated vessel with reflux of contrast with injection. Following treatment administration, another subtracted dual-phase CBCT was obtained from the same location.

### 2.3. CBCT Acquisition and Contrast Injection

Subtracted dual-phase CBCT was performed prior to embolization and after treatment (Innova 4100, GE Healthcare, Chicago, IL, USA). Non-contrast- and contrast-enhanced CBCTs were acquired during the same breath hold for each CBCT. The following acquisition parameters were used: 200° rotation angle at 40° per second leading to a total of 294 frames on a 40 × 40 cm detector with a 512 × 512 reconstruction matrix for the 3D volume. A long injection was used to achieve a steady state of contrast in the liver. At the steady state, the signal enhancement values for the parenchyma, the tumors, arteries and veins become independent of time [8]. A DSA was performed before the first CBCT to determine the appropriate delay for the acquisition. The time of delay was based on when optimal tumor blush was seen on DSA images. The same delay was utilized for all CBCTs achieved on the patient. The injection rate for the first CBCT was chosen by the operator according to the catheter or microcatheter location and the size of involved arteries. The injection rate for the pre-embolization CBCT was half the dose used in the DSA performed prior to the CBCT. After treatment, since part of the vasculature was embolized to complete stasis, the injection rate was lowered to half the rate that was used for the pre-embolization CBCT.

### 2.4. Calculation of Tumor Enhancement Parameters

***Pre- and Post-Embolization Adjusted Tumor Enhancement (ATE):*** Utilizing a prototype version of the Advantage Workstation (GE Healthcare, Buc, France), the pre-embolization non-contrast- and contrast-enhanced CBCTs were post-processed. A maximum of three distinct and well-defined tumors > 10 mm were identified in each patient. Semi-automatic contouring of the target tumor(s) was performed on the CBCTs, allowing for the quantification of the average contrast density within the tumor. To account for the impact of variability in contrast volume, concentration and injection rates on tumor enhancement, the contrast-enhanced vasculature (the main feeding artery to the tumor) was automatically extracted and used to adjust the signal of the supplied tumors. Therefore, the Adjusted Tumor Enhancement (ATE) was obtained by dividing the tumor enhancement value by the vasculature enhancement (ATE = Tumor enhancement/vasculature enhancement). Following embolization, a subtracted CBCT was also obtained and processed in a similar fashion to the pre-embolization CBCT. Subsequently, the difference between pre- and post-embolization ATE values were calculated, resulting in ∆ATE. The ∆ATE maps were computed using a prototype software.

***Post-embolization tumor enhancement fraction (PETEF):*** By analyzing the histogram of post-embolization CBCTs for each tumor and for background noise, the volume of the tumor that was still enhanced after embolization was determined. The PETEF was obtained by comparing this volume to the total volume of the tumor identified on the pre-embolization CBCT.

### 2.5. Assessment of Treatment Response

Treatment response was assessed using the 1 month follow-up CT or MRI. Two interventional radiologists independently evaluated the treatment response per modified Response Evaluation Criteria in Solid Tumors (mRECIST) criteria [9]. In cases of disagreement, a third interventional radiologist was involved to assess the treatment response. For analysis purposes, the response was grouped into a CR group and a non-CR group, which included partial response (PR), stable disease (SD) and progression of disease (PD) categories.

### 2.6. Local PFS and OS

L-PFS was defined as the time between the first embolization treatment and the detection of local progression at the site of treated tumor(s). OS was calculated from the time of first embolization treatment to time of recorded death. To account for the effect of multiple lesions per patient, we used a cluster data analytic method. Additionally, the tumor with the lowest ∆ATE in each patient was selected for L-PFS and OS analysis.

### 2.7. Statistical Analysis

The correlation between pre-embolization ATE, post-embolization ATE, ∆ATE, PETEF and tumor response was analyzed using the Mann–Whitney U test. Receiver operator curves (ROCs) were utilized to identify cutoff values. The Kaplan–Meier method was employed to calculate L-PFS and OS. Cox regression analysis was used to determine the influence of CBCT-based enhancement parameters on survival outcomes. Analysis was performed using Stata version 7 (StataCorp, College Station, TX, USA). *p* value of less than 0.05 was the threshold of significance.

## 3. Results

### 3.1. Patient Demographics

A total of 13 patients (mean age: 71, range: 33–84) with 29 tumors were included in this study. Table 1 demonstrates their demographic characteristics. Nine (69%) patients had cirrhosis with HCC (hepatitis C virus [n = 5, 55%], NASH [n = 1, 11%], hemochromatosis [n = 1, 11%], unknown [n = 2, 22%]). The remaining four (31%) patients had liver-dominate metastatic malignancies (three pancreatic neuroendocrine tumors, one salivary adenocarcinoma). All patients with cirrhosis were CP class A. ECOG performance status was 0 in all patients. A total of 15 treatment sessions were performed on the cohort. Most patients required a single treatment session (n = 11, 85%), while two patients with bilobar disease required two lobar treatments. Tumor involvement was multifocal in 61% (n = 8) of patients and solitary in 38% (n = 5). The mean diameter of treated tumor was 28 mm (range: 10–79 mm).

### 3.2. ATE and Treatment Response

**Figure 1:**
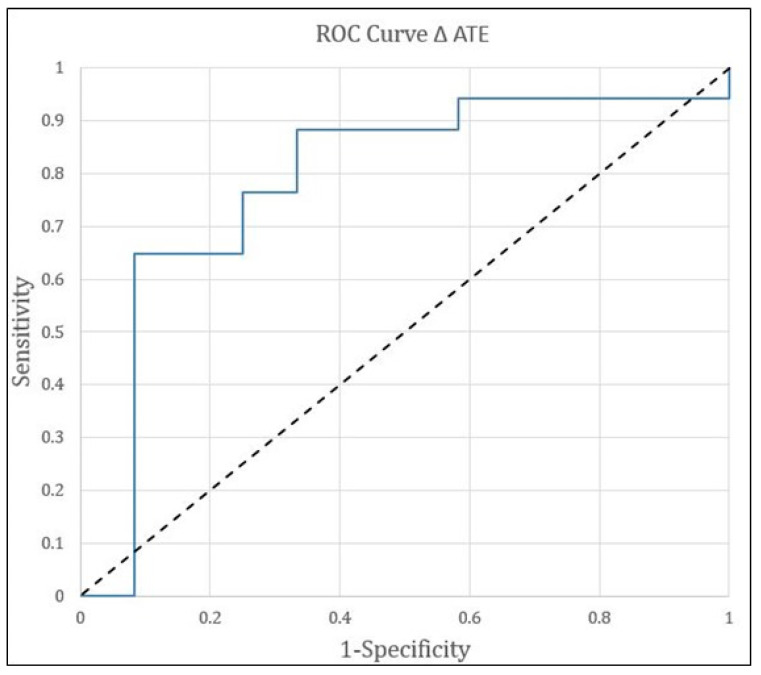
Receiver operator curve (ROC) of the correlation of ∆ATE with 1-month treatment response per mRECIST. A ∆ATE cutoff with 75% specificity and 76% sensitivity was chosen to differentiate the 2 groups. Progression-free survival and overall survival were calculated for patients with ∆ATE above and below this cutoff, corresponding to a ∆ATE value of 0.33.

A total of 29 tumors were analyzed. CR was seen in 17 (59%) treated tumors, and 12 (41%) treated tumors were in the non-CR group. The results are detailed in Table 2. Mean pre-embolization ATE in all tumors was 35 ± 11%. Pre-embolization ATE in the CR group was 39%, which was significantly higher compared to 30% in the non-CR group (*p =* 0.023). Mean post-embolization ATE in the CR group was 3.01% and 6% in the non-CR group (*p =* 0.156). Mean ∆ATE was 35 ± 10 percentage points. ∆ATE was 39 ± 10 percentage points in the CR and 29 ± 10 points in the non-CR group (*p =* 0.009). The bigger the ∆ATE or the bigger the decrease in enhancement following embolization, the stronger the correlation with CR (0.387 vs. 0.296, *p =* 0.009) (Figure 1).

### 3.3. PETEF and Treatment Response

**Figure 2:**
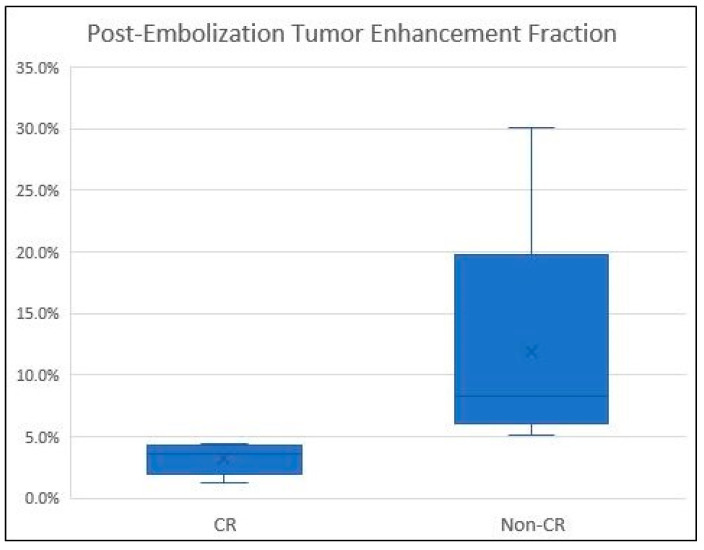
Post-embolization tumor enhancement fraction (PETEF) among HCC lesions. Tumors with PETEF < 5% consistently show complete tumor response (CR) per mRECIST at 1-month follow-up CT.

The overall PETEF was 3.7 ± 5.5% (Figure 2). PETEF was lower in the CR group (2.24%) compared to the non-CR group (6%); however, this difference was not statistically significant (*p* = 0.13). Subgroup analysis of the HCC tumors (11 tumors, 5 patients) demonstrated a lower PETEF among the CR group compared to the non-CR group (1.2% vs. 3.2%, respectively; Figure 2). In this subgroup, the difference was statistically significant (*p =* 0.004).

### 3.4. ATE and L-PFS and OS

**Figure 3:**
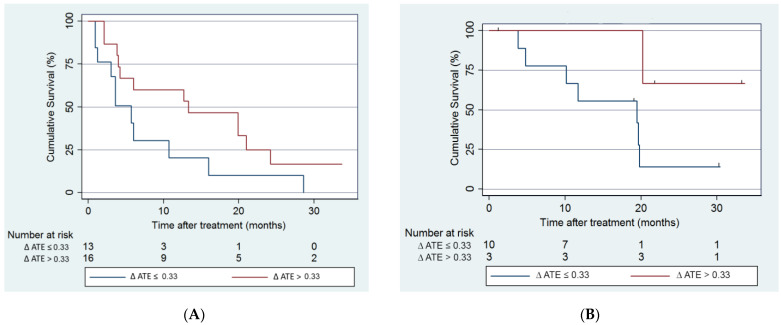
(**A**) Kaplan–Meier curve for progression-free survival (PFS). (**B**) Kaplan–Meier curve for overall survival (OS).

**Figure 4:**
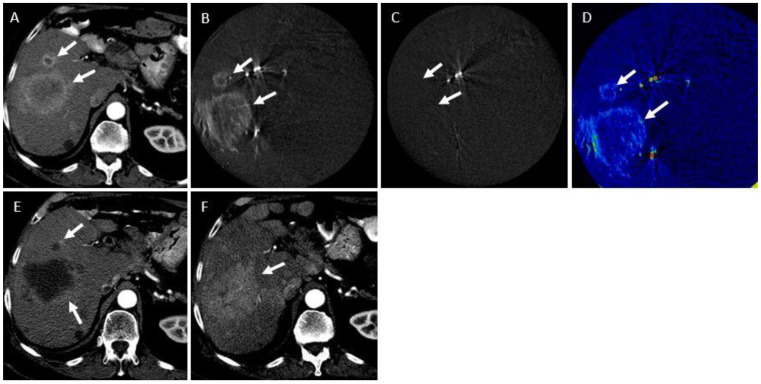
A 76-year-old male with hepatocellular carcinoma (HCC) presents for hepatic artery embolization. (**A**) Pre-procedural triphasic CT demonstrates two segment 8 lesions (7.6 cm and 1.7 cm in maximum axial diameter; arrows) with characteristic arterial-phase hyperenhancement, consistent with HCC. (**B**) Pre-embolization intraprocedural subtracted CBCT demonstrating the two hyperenhancing lesions corresponding to those on pre-procedural CT (arrows). (**C**) Post-embolization intraprocedural subtracted CBCT demonstrating complete absence of tumor enhancement in either lesion (arrows). (**D**) ∆ATE map demonstrates a ∆ATE of 20% for the large lesion and 23% for the smaller lesion. (**E**) One-month follow-up triphasic CT demonstrates residual areas of hyperenhancement on the arterial phase (arrow), which also wash out on the portal venous phase, consistent with residual HCC. Per mRECIST, treatment response was categorized as partial response (PR). (**F**) Three-month follow-up triphasic CT demonstrates local progression of disease (arrow).

**Figure 5:**
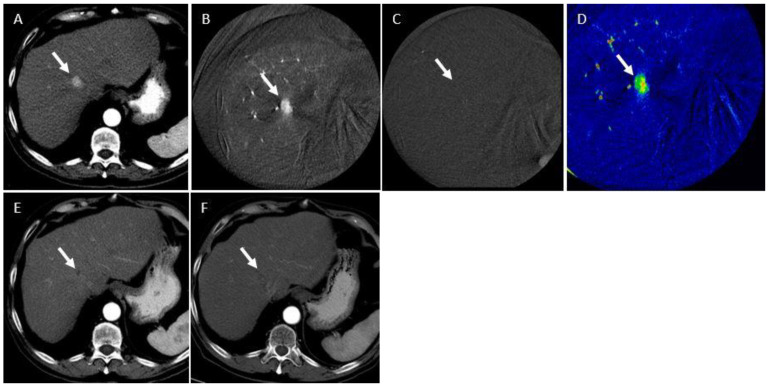
A 79-year-old male with hepatocellular carcinoma (HCC) presents for hepatic artery embolization. (**A**) Pre-procedural triphasic CT demonstrates one segment 8 lesion (1.8 cm in maximum axial diameter; arrow) with characteristic arterial-phase hyperenhancement, consistent with HCC. (**B**) Pre-embolization intraprocedural subtracted CBCT demonstrating the hyperenhancing lesion corresponding to the pre-procedural CT (arrows). (**C**) Post-embolization intraprocedural S-CBCT demonstrating complete absence of tumor enhancement (arrows). (**D**) ∆ATE map demonstrates a ∆ATE of 45%. (**E**) One-month follow-up triphasic CT demonstrates complete resolution of hyperenhancement on the arterial phase (arrow). Per mRECIST, treatment response was categorized as complete response (CR). (**F**) Three-month follow-up triphasic CT demonstrates no local progression or recurrence.

Median L-PFS for the entire group was 6 months. Using the cluster data analysis method, median L-PFS in the CR group was 12.7 months and 3.6 months in the non-CR group (*p =* 0.03). Median OS in the entire treated group was 19.6 months. Median OS in the CR group was 20.2 months and 11.6 months in the non-CR group (*p =* 0.043) (Figure 3). Figure 1 demonstrates a receiver operator curve (ROC), illustrating the association between ∆ATE and tumor response. A cutoff of ∆ATE at 33 points with sensitivity of 76% and specificity of 75% was selected to examine the association with local PFS. The median L-PFS of tumors with ∆ATE ≤ 33 points was 5.7 months (95% CI = 1.2–10.7), while the median L-PFS of tumors with ∆ATE > 33 was 13.1 months (95% CI = 3.8–21) (HR, 95% CI = 0.45, 0.20–0.9, *p =* 0.04). Based on the same ∆ATE cutoff of 33, patients having a tumor with the lowest ∆ATE ≤ 33 had a median OS of 19.7 months (95% CI = 3.77–19.8), while in the ∆ATE > 33 percentage points group, median OS was not reached (95% CI = 20.3–not reached) (HR, 95% CI = 0.15, 0.018–1.38, *p =* 0.04). Figure 4 demonstrates a patient with low ∆ATE and follow-up images demonstrate recurrence of disease. Figure 5 is an example of a high ∆ATE of 45, resulting in good response.

## 4. Discussion

In this study, subtracted dual-phase CBCT predicted initial response to HAE in patients with HCC or metastatic liver disease. CBCT-based enhancement in the tumor was adjusted and used to evaluate treatment response to HAE in patients with HCC or metastatic liver disease. A change of more than 33 points between the ATE before and after embolization was associated with both better L-PFS and OS. To our best knowledge, this is the first study assessing ΔATE and evaluating its role in predicting response and survival after HAE. This study supports previous studies reporting a correlation between enhancement parameters and tumor response. Loffroy et al. retrospectively analyzed 50 targeted HCC tumors in 29 patients treated with DEB-TACE [10]. They calculated the tumor enhancement on pre- and post-treatment dual-phase CBCT and reported a significant correlation between tumor enhancement on CBCT after TACE and complete and/or partial response in one-month follow-up imaging. Loffroy et al. measured the percentage of enhancement subjectively, unlike the current study, where this was performed objectively using a semi-automated method [10]. Borgheresi et al. investigated the value of tumor enhancement on pre-procedural triphasic CT [11]. They reviewed 63 patients with HCC treated with HAE and performed quantitative enhancement and perfusion measurements on the target tumor and the background liver on the triphasic CT performed before treatment. Unlike the current study, Borgheresi et al. observed that arterial enhancement did not predict immediate response or overall survival after embolization [2]. In the current study, higher pre-embolization ATE corresponded with better response to treatment.

The current study supports previous work reporting a correlation between perfusion variables and tumor response [12,13,14]. Vogl et al. measured changes in parenchymal blood volume (PBV) of malignant hepatic tumors in 111 patients treated with cTACE using a CBCT post-processing algorithm and reported that patients with initial tumor blood volume > 100 mL/1000 mL who demonstrated a decrease of 47.2% in blood volume had a 7.1% decrease in size on 4–6 weeks’ follow-up MRI, which was significantly higher compared to patients with lower decreases in blood volume [12]. In similar studies, de Korompay et al. and more recently Pellerin et al. investigated pre- and post-embolization PBV maps, respectively, in 40 patients with HCC undergoing bland embolization (n = 1), cTACE (n = 6) or DEB-TACE (n = 33) and 34 patients with metastatic colorectal cancer undergoing irinotecan drug-eluting bead chemoembolization (DEBIRI-TACE) [13,14]. Both studies demonstrated a significant correlation between a greater magnitude of decrease in median intratumoral blood volume (∆ PBV) and initial treatment response per mRECIST at 1-month CT/MRI follow-up [13,14]. In Vogl et al.’s study, the post-processing algorithm was based on cerebral venous blood volume in canines and, to our best knowledge, was not compared to CT-based PBV (gold standard) [15]. In both studies by de Korompay and Pellerin et al., a software was used for post-processing measurement. However, the method was not mentioned and the algorithm of converting signal to flow parameters was not described. Similar to prior studies, changes in perfusion or enhancement correlated with tumor response to treatment. In the current study, correlation between these variables and L-PFS and OS of the patients was also demonstrated.

PBV is a quantitative blood volume expressed in mL/100 mL which, in order to represent the blood volume, necessitates calibrations for each angiographic system to link pixel values extracted from the CBCT to gold-standard blood volume measurements, i.e., CT perfusion. This process can be challenging. ATE does not have these limitations. ATE is a unitless ratio between signal enhancements that does not need calibration and is simple to process based on a standard subtracted dual-phase CBC acquisition.

Another parameter that was evaluated in the current study is post-embolization tumor enhancement fraction (PETEF). The results of the HCC subgroup analysis suggest that a PETEF value ≤ 5% following embolization is predictive of CR (Figure 2). Although this analysis was conducted on a small number of tumors (n = 11), PETEF may be highly clinically relevant as a treatment endpoint for HCC embolization.

Two main confounding factors, including vascular anatomy and cardiovascular status, were identified prior to designing the study. In order to prevent them from impacting the outcomes, each patient’s parameters were compared to themselves before and after the injections. In other words, each patient served as their own control.

This study has multiple limitations, including a small sample size and retrospective methodology. Different tumor types were treated with HAE in this study; however, all tumors demonstrated arterial hypervascularity. Future studies with larger sample sizes are warranted to further examine the correlation between ATE and tumor response, PFS and OS. PETEF may also be clinically relevant as a treatment endpoint for HCC embolization and needs to be further investigated.

In conclusion, a greater decrease in ATE (∆ATE) following HAE is a predictor of treatment response, L-PFS and OS in patients with hepatic malignancies.

## Figures and Tables

**Table 1:** Demographic characteristics of treated patients.

Characteristics	Value
Sex	
Male	9 (69%)
Female	4 (31%)
Age (y, median, range)	71 (84–33)
Cirrhotic	
Yes	9 (69%)
No	4 (31%)
Etiology of Cirrhosis	
HCV	5
NASH	1
Hemochromatosis	1
Unknown	2
Type of Liver Malignancy	
HCC	9
Panc NET	3
Salivary adenocarcinoma	1
Child–Pugh Score	
A	13 (100%)
ECOG Performance Status	
0	13 (100%)
Previous Treatments	
No treatment	12
TACE	1
Pre-MELD Score (median, range)	11 (13–6)
Number of embolizations (n = 15)	
1	11
2	2
Lobar vs. Segmental	
Lobar	9
Segmental	4
Number of Tumors	
1	5
2	2
>3	6
Tumor Size (mm, median, IQR)	19 (15–31.5)

HCV = hepatitis C virus; NASH = non-alcoholic steatohepatitis; HCC = hepatocellular carcinoma; Panc NET = pancreatic neuroendocrine tumor; ECOG = Eastern Cooperative Oncology Group; TACE = transarterial chemoembolization.

**Table 2:** Tumor enhancement values in the treated patients.

All Tumors			HCC Only			Non-HCC		
Measured Parameters	Average Value (95% C.I)	*p*-Value	Tumors (n = 11)	Average Value (95% C.I)	*p*-Value	Tumors (n = 18)	Average Value (95% C.I)	*p*-Value
Pre-Embolization ATE								
All Tumors (n = 29)	0.350	n/a	0.328					
CR (n = 17)	0.385	0.023 *	CR (n = 6)	0.375	0.177	CR (n = 11)	0.426	0.101
Non-CR (n = 12)	0.304		Non-CR (n = 5)	0.274		Non-CR (n = 7)	0.326	
Post-Embolization ATE								
All patients (n = 29)	0.001	n/a	0.004					
CR (n = 17)	−0.003	0.156	CR (n = 6)	−0.001	0.329	CR (n = 11)	0.004	0.382
Non-CR (n = 12)	0.006		Non-CR (n = 5)	0.011		Non-CR (n = 7)	0.003	
∆ATE								
CR (n = 17)	0.387	0.009 *	CR (n = 6)	0.376	0.052	CR (n = 11)	0.393	**0.08**
Non-CR (n = 12)	0.296		Non-CR (n = 5)	0.261		Non-CR (n = 7)	0.321	
Post-Embolization Enhancement Fraction								
CR (n = 17)	0.022	0.128	CR (n = 6)	0.032	0.004 *	CR (n = 11)	0.017	0.659
Non-CR (n = 12)	0.060		Non-CR (n = 5)	0.120		Non-CR (n = 7)	0.017	

* Statistically significant *p* values (less than 0.05).

## Data Availability

The data presented in this study is available on request from the corresponding author.
